# AGE-RELATED DIFFERENCES IN THEORY OF MIND: A TIME-VARYING FUNCTIONAL CONNECTIVITY APPROACH

**DOI:** 10.1093/geroni/igac059.2080

**Published:** 2022-12-20

**Authors:** Samuel Naranjo Rincon, Richard Betzel, Anne Krendl

**Affiliations:** Indiana University at Bloomington, Bloomington, Indiana, United States; Indiana University, Bloomington, Indiana, United States; Indiana University, Bloomington, Indiana, United States

## Abstract

Theory of Mind (ToM) refers to the act of inferring someone’s inner state, such as their emotions and thoughts. Previous work also demonstrated that ToM performance declines across age, but the mechanisms underlying this are not well-understood. The brain regions underlying ToM are generally in the default mode network (DMN) – a group of brain regions particularly vulnerable to pathological aging. Recent work suggests that declines in functional connectivity (FC; correlations between brain regions) during resting state (a period of undirected thought) predict age deficits in ToM. However, these findings come from aggregated FC, ignoring potentially informative details of dynamic changes during resting state. Researching these details may provide more specificity related to social cognitive aging. To test this, 35 older adults (OA; M = 75.61; 22 female) and 40 young adults (YA; M = 21.58; 25 female) underwent resting state and task-based fMRI. During the task, they completed a standard ToM task. We conducted time-varying functional connectivity analyses in the DMN to identify dynamic changes over time. Overall, OA had more variability than YA, but variability benefitted YAs, and not OAs. This occurred specifically in a DMN subnetwork associated with contextual information in memory. These results suggest that variability is useful, but only to an extent. With respect to ToM, variability may promote how YA integrate contextual information to memory, but is ineffective for OA.

